# Improving Neurological Health in Aging Via Neuroplasticity-Based Computerized Exercise: Protocol for a Randomized Controlled Trial

**DOI:** 10.2196/59705

**Published:** 2024-08-08

**Authors:** Mouna Attarha, Ana Carolina de Figueiredo Pelegrino, Paule-Joanne Toussaint, Sarah-Jane Grant, Thomas Van Vleet, Etienne de Villers-Sidani

**Affiliations:** 1 Posit Science Corporation San Francisco, CA United States; 2 Department of Neurology and Neurosurgery Montreal Neurological Institute-Hospital McGill University Montreal, QC Canada

**Keywords:** brain training, cognitive training, healthy aging, neuroplasticity, acetylcholine, FEOBV, randomized controlled trial, aging, ageing, elderly, elder, older adults, older adult, neurological health, cognitive, computerized brain training, computerize, cognition, cognitive decline, Canada

## Abstract

**Background:**

Our current understanding of how computerized brain training drives cognitive and functional benefits remains incomplete. This paper describes the protocol for Improving Neurological Health in Aging via Neuroplasticity-based Computerized Exercise (INHANCE), a randomized controlled trial in healthy older adults designed to evaluate whether brain training improves cholinergic signaling.

**Objective:**

INHANCE evaluates whether 2 computerized training programs alter acetylcholine binding using the vesicular acetylcholine transporter ligand [18F] fluoroethoxybenzovesamicol ([18F] FEOBV) and positron emission tomography (PET).

**Methods:**

In this phase IIb, prospective, double-blind, parallel-arm, active-controlled randomized trial, a minimum of 92 community-dwelling healthy adults aged 65 years and older are randomly assigned to a brain training program designed using the principles of neuroplasticity (BrainHQ by Posit Science) or to an active control program of computer games designed for entertainment (eg, Solitaire). Both programs consist of 30-minute sessions, 7 times per week for 10 weeks (35 total hours), completed remotely at home using either loaned or personal devices. The primary outcome is the change in FEOBV binding in the anterior cingulate cortex, assessed at baseline and posttest. Exploratory cognitive and behavioral outcomes sensitive to acetylcholine are evaluated before, immediately after, and 3 months following the intervention to assess the maintenance of observed effects.

**Results:**

The trial was funded in September 2019. The study received approval from the Western Institutional Review Board in October 2020 with Research Ethics Board of McGill University Health Centre and Health Canada approvals in June 2021. The trial is currently ongoing. The first participant was enrolled in July 2021, enrollment closed when 93 participants were randomized in December 2023, and the trial will conclude in June 2024. The study team will be unblinded to conduct analyses after the final participant exits the study. We expect to publish the results in the fourth quarter of 2024.

**Conclusions:**

There remains a critical need to identify effective and scalable nonpharmaceutical interventions to enhance cognition in older adults. This trial contributes to our understanding of brain training by providing a potential neurochemical explanation of cognitive benefit.

**Trial Registration:**

ClinicalTrials.gov NCT04149457; https://clinicaltrials.gov/ct2/show/NCT04149457

**International Registered Report Identifier (IRRID):**

DERR1-10.2196/59705

## Introduction

### Background

Specific forms of computerized brain training demonstrate observable cognitive and functional benefits. The largest brain training trial to date, Advanced Cognitive Training for Independent and Vital Elderly (ACTIVE) [1], provides compelling evidence that speed of processing training yields enduring gains that generalize beyond the trained task. This speed training exercise called Double Decision (BrainHQ) has been associated with a 29%-48% reduction in the risk of dementia over a decade-long follow-up, depending on the number of training hours completed [2]. Additional studies Additional studies have reported a range of benefits, including decreased at-fault motor vehicle collisions [3], slowed decline in instrumental activities of daily living [4-8], greater likelihood of improved locus of control [9], improved self-rated health [10], reduced health care payer-related costs [11], decreased onset of age-related depression [12], alleviation of depressive symptoms [13], and a lowered risk of global decline in health-related quality of life [14,15].

Another form of training, called Freeze Frame (BrainHQ), was neuroscientifically designed to engage tonic and phasic attention to naturally upregulate neuromodulatory control and amplify the training benefits of Double Decision [16]. Initial investigations suggest notable improvements in executive function, skill acquisition, spatial and nonspatial attention, and sleep duration and quality [16-20].

The mechanisms underlying the cognitive benefits of brain training are largely unknown, although a few pilot studies, mostly conducted in clinical cohorts, offer intriguing insights. These studies suggest potential improvements in functional connectivity [21,22], improved brain synchronization [23], increased hippocampal activation [24], and improved white matter (WM) insulation between brain regions dedicated to visual and attentional processing [25]. Central to these network-based changes is synaptic plasticity, a fundamental process regulated by key neuromodulatory centers in the brain that oversee the release of neurotransmitters, most notably acetylcholine [26,27].

If age-related cognitive decline is paralleled by the atrophy and diminished signaling of acetylcholine, then it follows that augmenting acetylcholine may hold promise for enhancing cognition. A pilot investigation conducted at McGill University provides initial support for this view: a cohort of 5 healthy older adults engaged in a 6-week training program using Freeze Frame showed a 16%-24% increase in forebrain acetylcholine binding, which mirrored performance on a sustained vigilance assessment. Acetylcholine binding was measured through positron emission tomography (PET) using the radiotracer [18F] fluoroethoxybenzovesamicol ([18F] FEOBV) that binds to vesicular acetylcholine transporter [28]. Previous studies have demonstrated that FEOBV-PET binding patterns are consistent with the known organization of the cholinergic system and that aging is associated with a significant decline of 2.5% in FEOBV binding per decade in the anterior cingulate cortex [29].

### This Study

INHANCE is a phase IIb, prospective, double-blind, parallel-arm, active-controlled, randomized trial in healthy adults aged 65 years and older who are randomly assigned to a computerized speed and attention brain training program (BrainHQ) or to an active control of games designed for entertainment (eg, Solitaire).

The objective is to develop a mechanistic understanding of these training programs using FEOBV-PET. Exploratory endpoints include a standard cognitive battery (National Institutes of Health Executive Abilities: Measures and Instruments for Neurobehavioral Evaluation and Research [NIH EXAMINER]), behavioral assessments sensitive to acetylcholine (heart rate variability and pupillometry), and train-to-task assessments to evaluate target engagement. Both the cognitive and behavioral assessments are conducted at baseline, posttest, and at a no-contact 3-month follow-up to evaluate durability. We hypothesize that participants training on speed and attention will show greater FEOBV binding at posttest versus baseline within the anterior cingulate cortex compared with the active control. We do not consider the active control program to be fully inert and may find that both forms of training improve some (potentially different) indices of cognition performance and behavior.

## Methods

### Trial Design

Improving Neurological Health in Aging via Neuroplasticity-based Computerized Exercise (INHANCE) is a double-blind, parallel-arm, active-controlled, randomized clinical trial evaluating the superiority of speed and attention brain training against an active control of computer games.

### Recruitment and Eligibility

All participants were recruited near McGill University, Canada, where the FEOBV radiotracer is synthesized and administered.

Recruitment methods included public presentations (TV, radio stations, and conferences), newspapers, word of mouth, and flyers. Flyers were posted in churches, community centers, Facebook groups, local libraries, and at the neurology clinic at the Montreal Neurological Hospital. Flyers described the study and included information regarding inclusion and exclusion criteria as well as the method for contact. They described the opportunity to volunteer for a clinical trial to advance the science and treatment of age-related cognitive decline. Compensation was described in appropriate terms that were not overemphasized relative to the remainder of the text. No indication of “free medical treatment” was communicated. All materials used for advertising or recruitment received ethics approval before implementation.

Participants were screened for the following inclusion criteria: (1) potential participants must be 65 years or older at the time of study screening; (2) potential participants should be cognitively healthy and score ≥23 on the Montreal Cognitive Assessment (MoCA) [30]; (3) potential participants must be likely able to complete all primary outcome measures in the judgment of the investigator; (4) potential participants must demonstrate adequate decisional capacity, in the judgment of the investigator, and be capable of making an informed decision regarding their participation in this research study; (5) potential participants must have the visual, auditory, and motor capacity to use the computerized intervention in the judgment of the investigator; (6) potential participants must already have, be willing to obtain, or be willing to travel to locations with internet connectivity to complete intervention activities; and (7) potential participants must be able to communicate in either English or French.

Participants were excluded based on the following criteria: (1) potential participants have an existing diagnosis of major or minor neurocognitive disorder at screening; (2) potential participants answer “yes” to “active suicidal ideation” with “specific plan and intent” on the Columbia-Suicide Severity Rating Scale (C-SSRS) [31] or any of the suicide-related behaviors (actual attempt, interrupted attempt, aborted attempt, preparatory act, or behavior) on the “suicidal behavior” section if the ideation or behavior occurred within 2 months from participant’s date of consent (as recommended by the US Food and Drug Administration for treatment trials); (3) potential participants are depressed and score >10 on the Geriatric Depression Scale–Short Form (GDS-SF) [32,33]; (4) potential participants have been treated with a computer-based cognitive training program manufactured by Posit Science within 5 years of the date of consent given maintenance effects established in previous work [15]; (5) potential participants are participating in a concurrent clinical trial (involving an investigational pharmaceutical, behavioral treatment, medical device, or other) that, in the judgment of the investigator, could affect the outcome of this study; (6) potential participants are pregnant or breastfeeding due to unknown effects of the FEOBV radioligand in pregnant and nursing individuals; (7) potential participants have claustrophobia or implantation with any medical devices above the waist that may concentrate radio frequency fields or have other medical issues that may frustrate participation in magnetic resonance imaging (MRI) procedures; (8) potential participants have medical illnesses deemed to interfere with participation in study activities or unstable or untreated conditions that may affect cognition, including substance abuse or dependence disorders, drugs that interfere with cholinergic function, ongoing chemotherapy, or other cancer treatment; and (9) potential participants show signs of intoxication due to current substance abuse (including alcohol or illegal drugs).

### Randomization

Given the potential importance of demographic variables in response to training, we used a minimization method of adaptive stratified randomization with a 1:1 allocation; the “platinum standard” of randomization methods when stratification is required [34] to minimize the imbalance between the number of participants in each group over factors known to influence performance. We balanced groups based on the distribution of acetylcholine scores and the NIH EXAMINER composite score. Previous research has shown that binding in the anterior cingulate significantly decreases with age while binding in the occipital cortex does not [29]. The acetylcholine score is defined as the ratio of the anterior cingulate to the occipital cortex to mitigate interindividual differences in baseline blinding.

The minimization method addresses the general problem of ensuring stratification across multiple factors in small- or moderate-sized trials. The various imbalances are added together to give the overall imbalance in the study. Group assignment is then made at random with a heavy probability weighting (0.8) in favor of the group that would minimize imbalance. Out of 2, 1 participant (50%) will be assigned to the intervention group, and 1 participant (50%) will be assigned to the active control group.

**Figure 1:**
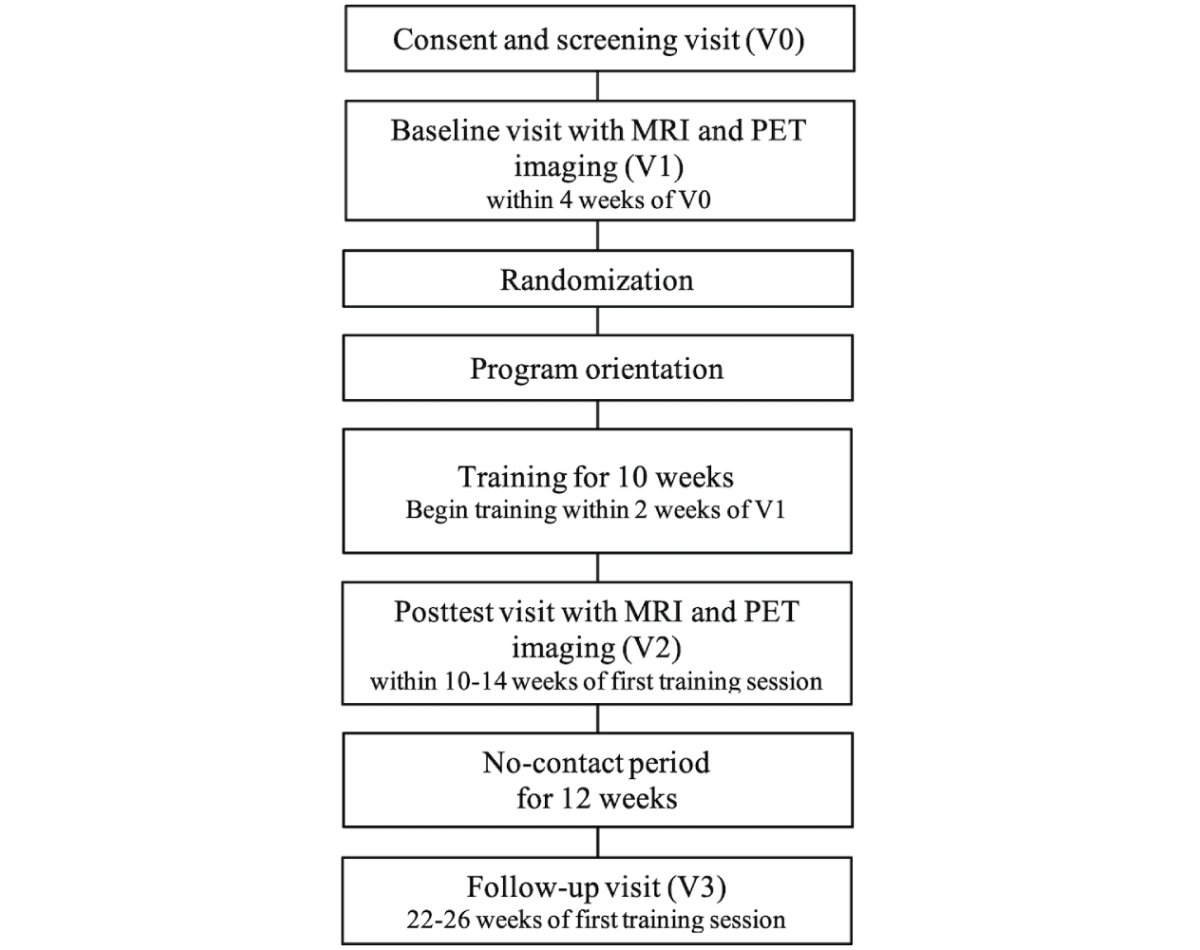
Study flow diagram. MRI: magnetic resonance imaging; PET: positron emission tomography.

Participant allocation was performed after the baseline visit (V1) and before the planned first day of program use. Baseline data were fully monitored for each participant, with all queries resolved, because allocation depended on obtaining data for the 2 prognostic variables. See the study flow diagram in Figure 1.

#### Consent and Screening Visit (V0)

Study staff will discuss study goals, activities, and requirements with the potential participant, complete the informed consent discussion, and if appropriate the potential participant will consent to join the study. Study staff will perform required inclusion and exclusion assessments. This visit will last approximately 1 hour.

#### Baseline Visit (V1)

If the participants are eligible, the participant will complete the cognitive assessment battery and the pretraining PET and MRI recordings. This visit will last approximately 3 hours and can be split into 2 sessions if needed.

#### Randomization

The participants will be randomized after the baseline visit and before the planned first day of the training program. Randomization will not take place until the screening and baseline data have been fully reviewed, residual data queries have been resolved, electronic data capture fields have been monitored, and it has been verified that the participant has met eligibility requirements.

#### Program Orientation

The participants will attend a program orientation session so that study staff may orient them to the training program in person or remotely. The participant will also receive detailed written information on how to perform the training program over the next several weeks. During this period, study staff will conduct weekly check-ins over the phone or email to answer questions and troubleshoot issues.

#### Intervention Period

The participants will engage in the assigned program for approximately 30 minutes per session, 7 sessions per week, for 10 weeks (approximately 3 months after randomization is complete).

#### Posttest Visit (V2)

The cognitive assessment battery along with PET and MRI imaging will be completed following the intervention period, approximately 3 months after initial enrollment. The participants will be informed that they will no longer have access to the intervention applications after this visit. This visit will last approximately 3 hours and may be split into 2 sessions if needed.

#### No-Contact Period

After the posttest visit, the participant will be entered into a follow-up period lasting 3 months with no further program use. Study staff may connect with the participant once a month during this time to maintain engagement.

#### Follow-Up Visit (End of Study, V3)

This assessment visit will be completed approximately 6 months after the initial enrollment. The participants will complete the cognitive assessment battery and will be formally exited from the study following this visit, concluding their participation. This visit will last approximately 1.5 hours.

### Blinding

Unblinded study staff will provide support for participants using their assigned programs. Only blinded study staff are authorized to participate in assessment administration, scoring, evaluation, follow-up of study participants after randomization, and baseline data analyses. The principal investigators and site investigator are required to complete a Delegation of Authority Form before the start of the study indicating which activities individual site research team members will be authorized to complete. To prevent unblinding, the following controls occurred at the site level: (1) the cognitive training condition and the active control condition will be identified as “Treatment A” and “Treatment B”; (2) participants will be instructed and reminded not to discuss details related to their training program with site staff, colleagues, friends, or acquaintances; (3) principal investigators will be instructed to not discuss details of either treatment arm with site personnel; (4) the site will be required to minimize the possibility of accidental unblinding of the site staff (eg, unintended viewing of treatment sessions); and (5) signage will be posted in appropriate areas throughout the facility reminding participants to not discuss study details.

### [18F] Fluoroethoxybenzovesamicol

PET imaging will be performed with a radioligand, FEOBV [35-38], which will be administered intravenously through a fine-needle catheter inserted into an arm vein. Participants will wait for approximately 180 minutes for FEOBV to distribute across the brain. During this time, they will remain at rest but will be able to use the washroom and walk around if desired. The expected dose for 2 PET scans is estimated at 11-15.4 mSv and does not exceed the nationally accepted limits of 50 mSv per year [37]. FEOBV is undetectable at 20 hours post administration.

Participants will be positioned lying on their backs for the PET imaging session. A transmission study lasting 5 minutes will be performed using a 68 Ge source. Participants will then receive a slow push intravenous injection of 350-400 MBq of FEOBV (which corresponds to 8.05 mSv to 9.2 mSV given that the dose per MBq of FEOBV is 0.023 mSv per MBq). A 60-minute list mode acquisition will be launched at the start of the injection. The PET marker will be produced according to methods described previously [39]. All PET imaging sessions will be supervised by a qualified nuclear medicine physician.

### Intervention

**Figure 2:**
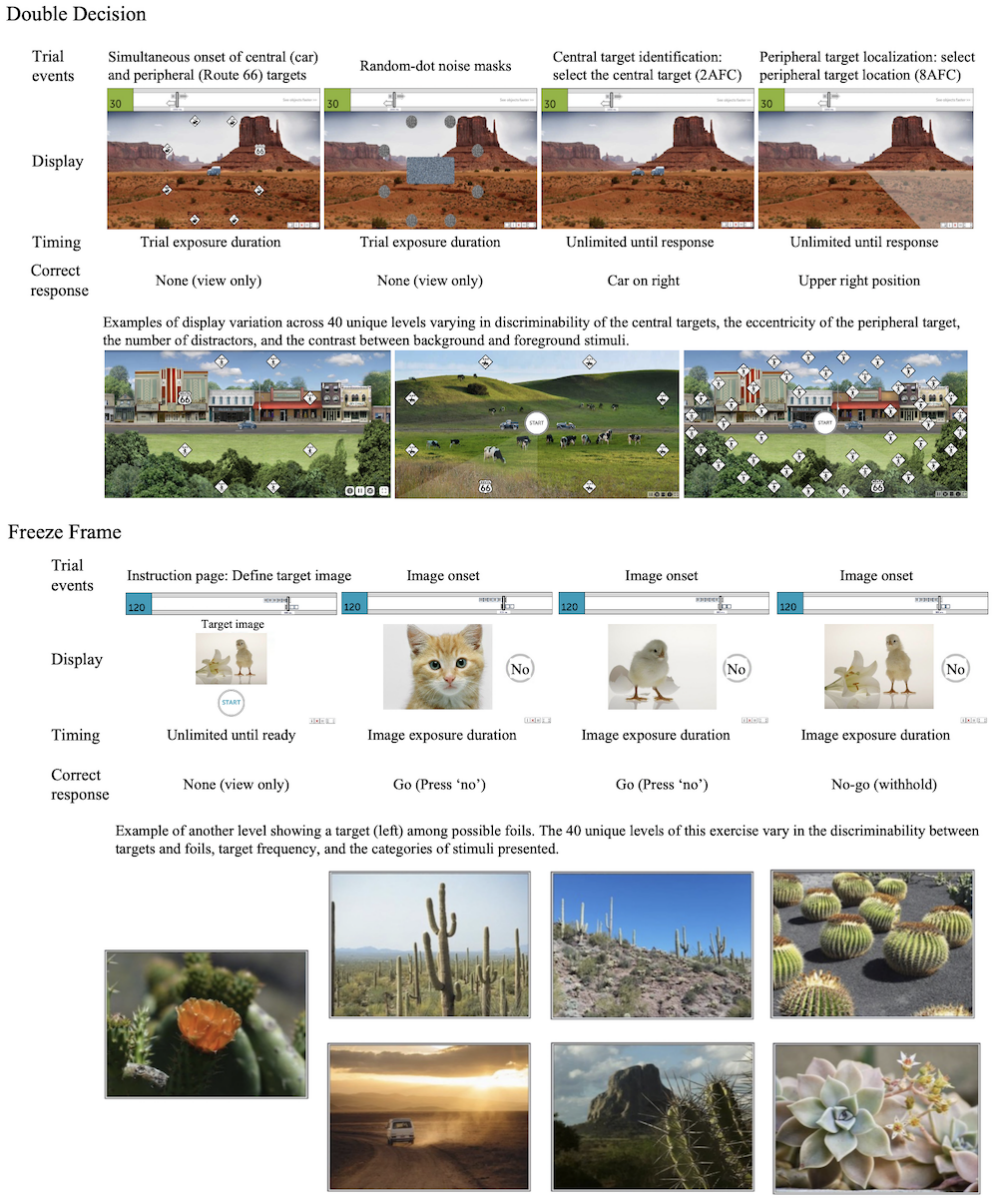
Trial events for Double Decision and Freeze Frame.

The intervention included Double Decision [2,5,40] and Freeze Frame [16,17,20], designed using the principles of neuroplasticity, in a 35-hour training schedule delivered over a 10-week period (approximately 30 minutes per session, 7 sessions per week, for a total of 70 sessions). All training was completed remotely at the participant’s home. See Figure 2 for the trial events of both exercises.

Double Decision trains visual speed of processing and selective attention [2,5,40]. In this dual-task exercise, participants discriminate a visual stimulus presented in the center of gaze while simultaneously locating a target in the peripheral visual field. There are 40 unique levels that vary in the discriminability of the central targets, the eccentricity of the peripheral target, the contrast between background and foreground stimuli, and the number of peripheral distractors. The adaptive dimension is display exposure duration (how long the items are on the screen) [41]. As the participant gets trials correct, exposure duration decreases requiring less time to complete the task; conversely, as the participant gets trials incorrect, exposure duration increases to make the task easier. Raw scores are defined as the exposure duration at approximately 80% criterion accuracy in milliseconds. The best possible raw score is 32 milliseconds and the worst possible score is 3162 milliseconds. Analyses will use *z* scores generated using the mean and SD from the intent-to-treat (ITT) population.

Freeze Frame trains 2 well-characterized, intrinsic properties of neuromodulatory control, which are tonic and phasic alertness [16,17,20]. Tonic alertness refers to the ongoing state of intrinsic readiness that fluctuates in the order of minutes to hours and is intimately involved with sustaining attention while providing the cognitive tone necessary for performing more complicated functions such as working memory and executive control. In contrast, phasic alertness is the rapid modulation in alertness due to any briefly engaging event and is vital for operations such as orienting and selective attention. In this continuous performance training exercise, participants must remember a target image presented at the start of the trial after which a continuous stream of targets and foils are interleaved with unequal probability. Users must withhold the prepotent motor response to all targets and respond to only foils. The 40 unique levels vary in the discriminability between targets and foils, the speed at which the images are presented, and the categories of the stimuli presented. The adaptive dimension is target and foil frequency defined as the single target frequency required for users to achieve at least 80% accuracy (n=24/30) in target identification and 80% (n=24/30) in foil identification within each epoch of 30 trials [42]. The adaptivity range is 1-7 corresponding to 40% (12/30 trials), 35% (10-11/30 trials), 30% (9/30 trials), 25% (7-8/30 trials), 20% (6/30 trials), 15% (4-5/30 trials), and 10% (3/30 trials) target frequencies, respectively. Scoring is available as 1-7, overall mean accuracy, and d’prime as a measure of sensitivity. Higher scores indicate better performance. Analyses will use *z* scores generated using the mean and SD from the ITT population.

### Active Control

**Figure 3:**
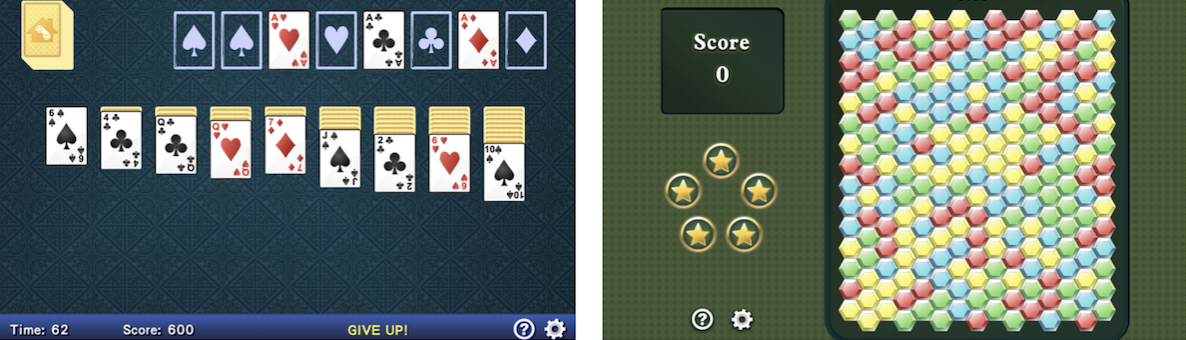
Games used in the active control arm include spinoffs of solitaire (left) and Bricks Breaker (right).

The active control includes previously vetted and popular casual video games designed for entertainment for a 35-hour training schedule delivered over a 10-week period (30 minutes per session, 7 sessions per week, for a total of 70 sessions). Games are rated E (for everyone) by the Entertainment Software Rating Board. All training was completed at the participant’s home. See Figure 3 for displays of these well-known games.

We matched the intervention and active control in overall program use intensity, visual modality, staff interaction, and engagement to provide a comparison group that matches the intervention on the aforementioned attributes without the key elements specific to improving neuromodulatory control. The games are designed to be nonspeeded, nonadaptive, and to not actively engage neural systems that underlie aging.

Games will be accessed by participants on the same platform as participants in the intervention to minimize unblinding.

### Sample Size

Based on the 0.27 SD seen in anterior cingulate FEOBV binding from our pilot study, detecting a small-moderate effect size (17% increase in binding in anterior cingulate FEOBV binding postintervention) with a power of 0.8 and α *P* value of .05 would require 40 participants in each of the 2 groups (80 total). We assume a 15% attrition rate and will enroll at least 92 participants to achieve 80 completers.

### Ethical Considerations

The study has been developed in accordance with the Declaration of Helsinki guidelines and was approved by the Western Institutional Review Board (IRB00000533) and the Research Ethics Board of McGill University Health Centre (2020-6474). The radioligand 18F-FEOBV was approved by Health Canada (control # 252085).

### Informed Consent

Prospective participants will undergo a phone screen that includes an overview of study details and a discussion of risks along with questions to assess the inclusion and exclusion criteria. Eligible individuals expressing interest in the study will be invited to the study site to complete the informed consent discussion, followed by a behavioral screening session, health questionnaire, and interview. The informed consent discussion will take place in a private room at the study site. Participants will be provided a copy of the approved consent form detailing the general purposes and procedures of the study. Study staff will provide detailed information about study requirements, offer participants an opportunity to ask questions, and provide clarification before signing the consent. Participants will be encouraged to take time to consider whether they wish to participate in the study and discuss with family before signing the consent. At the participant’s request, study staff may be asked to step out of the room to provide privacy for such discussions. To participate in this study, an individual must be judged capable of understanding the nature of the research, risks, and potential benefits. The consent form will clearly state that the participant may withdraw consent and stop participation at any time without judgment or penalty. Potential risks and benefits will be explained by the study staff, and the participants will be asked to sign the consent form. A member of the study staff will also sign the consent form. No study activities will take place before participant consent.

On the consent form, both the treatment and active control programs will be described as “computer-based activities designed to engage cognitive processing,” and the goal of the trial will be described as “comparing 2 forms of cognitive remediation.” The goal of this description is to ensure that if participants realize that they are engaged in a training experience that differs from other friends or colleagues enrolled in the trial, they will not assume that they are participating in an experimental versus control, thereby maintaining the integrity of the blinded study.

The consenting study team member will inform participants about compensation for their participation in the study. We will provide US $30 (CAD $40) for the baseline visit and PET and MRI imaging (V1), US $10 (CAD $13) for every 10 training sessions completed (maximum of US $70 [CAD $91] for completing all 70 sessions) during the intervention period, US $30 (CAD $40) for completing the posttest visit and PET and MRI imaging (V2) and US $30 (CAD $40) for the end-of-study 3-month follow-up visit (V3). Participants who complete the study in its entirety will be reimbursed US $160 (CAD $211). Payment for assessment visits will occur following the completion of that visit. If a participant must repeat an assessment visit due to administrative assessment or study staff errors, participants may be provided additional compensation for that session. If the participant does not complete the study or withdraws early for any reason, the participant will only be compensated for the study visits and training sessions they have completed.

Participants will either be given a loaner tablet (Lenovo M10 Android 9.0 tablet) to complete their assigned training exercises to help bridge gaps in digital access or be given the option to train on a personal device.

One fully executed copy of the informed consent will be provided directly to the participant, and the original signed form will be retained in a secure manner at the study site and be available for inspection at the study site upon the request of representatives of relevant regulatory agencies. The copy of the consent form taken home by the participant will include appropriate telephone and email contact information, including the site investigator and the site’s reviewing IRB.

Spouses and partners of participants are not part of INHANCE but, if interested, will receive a complimentary license to the same training program as the participant to reduce the risk of shared training on the same account.

### Measures and Data Collection

See Table 1 for all measures and time points.

**Table 1:** List of study activities and time points used in Improving Neurological Health in Aging via Neuroplasticity-based Computerized Exercise (INHANCE). Unanticipated adverse device effects were reported as needed. Study exit was reported when the participants exited from the study.

Study activity	Consent and screening visit (V0)^a^	Baseline visit (V1)^b^ within 4 weeks of V0	Program orientation and intervention for 10 weeks begin training within 2 weeks of V1	Posttest visit (V2)^c^ within 10-14 weeks of the first training session	No-contact period for 12 weeks	Follow-up visit (V3)^d^ within 16-22 weeks of the first training session
Informed consent	✓					
Inclusion and exclusion criteria	✓					
Demographics	✓					
Medical history	✓					
Medications	✓					
MoCA^e^	✓					
GDS-SF^f^	✓					
C-SSRS^g^, baseline	✓					
MRI^h^ and PET^i^ imaging		✓		✓		
NIH EXAMINER^j^		✓		✓		✓
Train-to-task assessments with heart rate variability and pupillometry		✓		✓		✓
C-SSRS, since last visit		✓		✓		✓
Medications, since the last visit		✓		✓		✓
Adverse effects		✓		✓		✓
Randomization		✓				
Program Orientation			✓			
Computer training			✓			
Weekly phone check-in			✓			

^a^V0: consent and screening visit.

^b^V1: baseline visit.

^c^V2: posttest visit.

^d^V3: follow-up visit (end of study).

^e^MoCA: Montreal Cognitive Assessment.

^f^GDS-SF: Geriatric Depression Scale–Short Form.

^g^C-SSRS: The Columbia Suicide Severity Rating Scale.

^h^MRI: magnetic resonance imaging.

^i^PET: positron-emission tomography.

^j^NIH EXAMINER: National Institutes of Health Executive Abilities: Measures and Instruments for Neurobehavioral Evaluation and Research.

### Screening

Following informed consent, potential participants will complete a set of structured interviews, neuropsychological assessments, and self-report questionnaires to evaluate their eligibility for the study. The following measures will be administered to participants at screening: (1) A structured clinical interview (20 minutes, in person) will collect key demographic (eg, year of birth, age, and education) and medical history information, including medical diagnoses and current medications. (2) The MoCA (10 minutes, in person) will assess global cognitive function. The MoCA tests visuospatial processing, executive functioning, naming, memory, attention, language, abstraction, delayed recall, and orientation to time and place for a total of 30 points, with higher scores indicating better performance [43]. Potential participants must score 23 or above on the MoCA to be eligible for INHANCE. This cutoff maximizes true positives and minimizes false positives (Youden index=0.71) [30]. Those who receive a score lower than 23 are referred to a neurologist for an evaluation of cognitive impairment. (3) The GDS-SF (7 minutes, in person) will assess depression. The GDS-SF is a self-report questionnaire in which participants respond with a yes or no to 15 questions about how they felt over the past week [32,33]. This assessment has good internal consistency (α coefficient of .82) and validity (dimensionality coefficients of .94 across multiple time points) [44,45]. Potential participants must score 9 or less on the GDS-SF to be eligible for INHANCE [45]. Those who receive a score of 10 or higher are referred to a physician for appropriate treatment. The Columbia Suicide Severity Rating Scale (C-SSRS) (10 minutes, in person) will assess suicidal ideation and behavior [31]. The C-SSRS is a questionnaire with high sensitivity and specificity that assesses the severity of ideation, intensity of ideation, suicidal behavior, and lethality [31]. Potential participants who endorse suicidal ideation (question 5 “Active suicidal ideation with specific plan and intent”) or any suicide-related behaviors (actual attempt, interrupted attempt, aborted attempt, preparatory act, or behavior) if the ideation or behavior occurred within 2 months from participant’s date of consent will be referred to a physician for appropriate treatment. In addition, study participants meeting these criteria at any timepoint throughout the study will be asked to complete a final assessment, if appropriate, and then withdraw from the study to be referred for appropriate treatment.

### Outcome Measures

Outcome measures will assess the extent to which brain training leads to measurable changes within the cholinergic system, cognition, and behavior in healthy older adults. The primary outcome is acetylcholine binding measured by FEOBV-PET [37,38] at baseline versus posttest. All other outcomes are exploratory and will be measured at baseline, posttest, and at a 3-month follow-up after the training is complete. (1) A structural MRI (20 minutes, in person) will be acquired during the first scanning session to coregister the PET data with each individual’s neuroanatomical brain image. In total, 3-dimensional T1-weighted anatomical MR image volumes covering the entire brain will be acquired on a 3T Siemens Magnetom Prisma (Siemens) scanner with an 8 channels head coil (repetition time=27 ms; echo time=9.20 ms; between 176 and 192 sagittally oriented slices with a slice thickness of 1 mm; acquisition matrix=240×256; field of view=256 mm). (2) The National Institutes of Health Executive Abilities: Measures and Instruments for Neurobehavioral Evaluation and Research (NIH EXAMINER; 20 minutes, in person) will assess cognitive performance [46]. Participants will complete a set of computerized assessments evaluating executive function (Flanker, Set-Shifting, and Anti-Saccades) and performance will be measured using the composite *z* score shown to have good reliability and validity [47]. (3) Behavioral assessments of heart rate variability and pupillometry will assess cholinergic function (15 minutes, in person). Pupillometry acquisition will be carried out using Tobii Pro Glasses 2 (Tobii) and heart rate variability will be acquired using a 4-lead Consensys ECG Development Kit (Shimmer). Both are measured while completing the 2 train-to-task assessments described below. (4) Computerized train-to-task assessments for Double Decision and Freeze Frame (15 minutes, in person) will serve as a positive control for task learning [48]. We expect large improvements in the intervention group on these assessments because they have directly practiced these tasks. The data are relevant because individuals failing to make progress on train-to-task assessments may represent a subpopulation not treatable with this program, and individuals making strong progress may represent a subpopulation particularly amenable to treatment with this program. The 2 assessments in this group are modeled upon a single level from their respective training exercise using a scoring algorithm that waits until the participant achieves asymptotic performance.

### Data Management

All study-related data are recorded into a secure, web-based electronic case report form at the study site through the Longitudinal Online Research and Imaging System (LORIS). This system meets all relevant privacy and security standards for electronic clinical trial data entry and storage, as well as the Health Insurance Portability and Accountability Act and Personal Information Protection and Electronic Documents Act standards for confidentiality and privacy [49].

Following consent, each participant will be assigned a standardized Participant Identification Number composed of digits to identify the study and digits to identify the participant. All electronic case report form data entry will be deidentified, using the Participant Identification Number and not the participant’s name, but will include identifying information in the form, such as the date of the assessment administration, age, and potentially other dates associated with the data collected.

### PET Image Processing

The SPM12 software [50] is used to perform all data preprocessing according to the following steps: (1) the T1-weighted structural images of all participants are spatially normalized to the MNI-152 standard reference template [51], and segmented into gray matter, WM, and cerebrospinal fluid tissues using Diffeomorphic Anatomical Registration Through Exponentiated Lie Algebra (DARTEL) algorithm [52]; (2) the static FEOBV PET images are then coregistered to the participant’s T1, and from there to the MNI-152 template by applying the deformation fields obtained in the first step. A Muller-Gartner partial-volume correction method is used to remove the partial volume effect on the PET images [52]; (3) finally, normalized partial volume effect–corrected FEOBV PET images are spatially smoothed to 8 mm full width at half maximum to remove random noise.

For all spatially normalized FEOBV PET images, the mean standard uptake value ratios are computed in preselected regions of interest (ROIs) using a supratentorial WM mask as a reference region to normalize the FEOBV PET images [53]. An anatomical MNI-space atlas (Hammers Atlas) [54] is applied to the PET images to quantify regional differences in FEOBV uptake within preselected ROIs.

### Statistical Analysis

The data analysis plan defines a primary ITT population, a single primary outcome measure, a set of exploratory outcome measures, a single primary evaluation time point, an exploratory evaluation time point, a primary statistical analysis methodology, a criterion for statistical significance, and guidance for the interpretation of results.

The primary ITT population is defined as all participants who were randomized and completed the first training session [55]. This includes all randomized participants except those who drop or withdraw post randomization and pretraining.

The primary outcome measure assesses forebrain FEOBV binding in the anterior cingulate cortex at baseline and posttest between the intervention and active control groups. The exploratory outcome measures are FEOBV binding across additional ROIs (posterior anterior cingulate, primary auditory cortex, primary sensorimotor cortex, parietal lobe, frontal lobe, occipital lobe, temporal lobe, global cortex, hippocampus, parahippocampal gyrus, putamen, caudate, striatum, nucleus basalis of Meynert), NIH EXAMINER composite score, Double Decision and Freeze Frame train-to-task assessment scores, heart rate variability, pupillometry, and MRI volumetrics. The 3-month follow-up assessment is an exploratory timepoint.

The primary statistical analysis methodology is a linear mixed model approach. We will first compare intervention and active control groups in the ITT population at baseline using *t* tests or chi-square tests to determine if differences in baseline variables remain after the randomization process. Variables that show a significant α (*P*<.05) will be noted and used as covariates in the model [55]. We will examine the data for each outcome measure using a linear mixed effects model with treatment group and time as fixed factors, and covariates as necessary from the baseline analysis. Missing data will be accounted for using iterative full-information maximum likelihood estimation. Within-group effects for each time point (posttraining, follow-up) will be calculated using data from each group. Between-group effects for each time point will be calculated in the same way, adding an interaction term (training group × time) to estimate the effect of cognitive training on outcome measure change. *P* values and effect sizes will be reported.

To evaluate the efficacy of BrainHQ to upregulate acetylcholine, we will conduct the analysis based on the baseline and posttest data. Finding a significant α of *P*<.05 on the FEOBV primary outcome measure will support the statement that the intervention increases acetylcholine binding.

To evaluate the endurance of cognitive effects following the completion of training (NIH EXAMINER), we will conduct confirmatory analyses based on the posttest and follow-up assessment data to evaluate the endurance of the change (maintenance, decline, or increase).

Showing maintenance or improvement between posttest and follow-up will support the statement that a brief intensive training epoch maintains (or improves) cognition after training has ceased.

Additional exploratory analyses will use Pearson or Spearman correlations [56] as relevant and will compare FEOBV binding against the NIH EXAMINER composite score, all exploratory ROIs, the train-to-task assessments, heart rate variability, pupillometry, MoCA performance, demographic variables, and the number of levels trained for the ITT. We will also conduct these analyses for those who completed the minimum effective dose of 10 hours of training or achieved a score on the Double Decision posttest assessment of 400 milliseconds or faster [1,57-59]. We will compare the NIH EXAMINER composite score with the primary and exploratory ROIs, train-to-task assessments, heart rate variability, pupillometry, MRI volumetrics, MoCA, and the number of levels trained for the ITT, and for those who completed the 10-hour minimum and achieved 400 milliseconds or faster on the Double Decision posttest assessment. Responder analyses will evaluate key moderators (age, sex, MoCA, baseline cognitive performance on NIH EXAMINER composite, baseline cognitive performance on the train-to-task assessments, and number of levels completed) to assess who responds most to training. No correction for multiple comparisons will be made for exploratory analyses and all trending relationships (P<.10) will be reported.

## Results

### Overview

The trial was funded in September 2019. The first participant was enrolled in July 2021, enrollment closed when 93 participants were randomized in December 2023, and the trial will conclude in June 2024. The trial is currently ongoing. The study team will be unblinded to conduct analyses after the final participant exits the study. We expect to publish the results in the fourth quarter of 2024.

### Dissemination

In accordance with the NIH Policy on Dissemination of NIH-Funded Clinical Trial Information (NOT-OD-16-149) and the Clinical Trial Registration and Results Information Submission regulation (42 CFR Part 11), INHANCE was registered on ClinicalTrials.gov within 21 calendar days after the enrollment of the first participant, and the study page has been continuously maintained and meets all reporting and regulatory requirements, and the study registration link was shared on all participant consent forms.

Data from this trial will be broadly shared with the research community and laypersons through the conventional scientific publication process. The final study results will be submitted to a peer-reviewed indexed scientific journal within 2 years after the last participant’s final study visit. Following the completion of the a priori data analysis plan, we will make the complete data set available to McGill investigators to conduct further post hoc exploratory analyses. Any relevant results will be submitted to relevant peer-reviewed journals. In 2026, two years after the completion of the trial, the complete raw data set will be available in LORIS, a freely available, open-source, and provenance-sharing data archive.

If the intervention shows a positive benefit on the primary outcome measure, then global distribution of an effective, low-risk, highly scalable brain training program will be justified and INHANCE will be commercially available on popular web-based platforms (eg, Chrome, Safari, and Firefox), iOS (Apple Inc) and Android devices across 14 languages (English, French, Spanish, Japanese, German, Hebrew, Italian, Korean, Portuguese, Greek, Dutch, Arabic, Mandarin, and Russian).

## Discussion

### Brief Summary

The goal of INHANCE is to conduct a double-blind, parallel-arm, active-controlled randomized trial to evaluate the impact of a 10-week computerized brain training program focused on speed and attention versus active control on cholinergic signaling in healthy adults aged 65 years and older, on the path to understanding how brain training enhances cognition. Recruitment for the trial began in July 2021 and concluded in December 2023, with the study set to complete in June 2024. Should the intervention demonstrate an increase in acetylcholine binding at posttest compared with baseline, the trial would identify a core mechanism of action and support the adoption of a simple, cost-effective, and scalable form of computerized training to protect against age-related cognitive decline.

### Comparison to Existing Work

Acetylcholine is widely recognized as a crucial neurotransmitter that regulates synaptic plasticity and supports overall brain health. Despite this, in vivo measurement of acetylcholine in humans has been scarce [36,60]. FEOBV-PET provides unique insights into the cholinergic system’s effect on cognitive performance, and this current trial is the largest imaging study of this radioligand to date. The trial’s findings will enhance our understanding of FEOBV’s association with aging and cognitive performance and its sensitivity to behavioral intervention.

### Limitations of This Study

Both training programs required a substantial time commitment of 30 minutes per session, 7 days a week for 10 weeks, totaling 35 hours. While missed sessions are anticipated, the frequency and total training hours surpass those in previous studies and meta-analyses of brain training [40,61]. In addition, both interventions require participants to operate an internet-connected device (or be willing to learn), which may limit the generalizability of the recruited sample.

### Conclusions

Given the widespread interest and ongoing initiatives to identify nonpharmacological interventions for preventing and treating cognitive decline (eg, World-Wide FINGER trials [62-67]), the aims of the current study are of great importance: to further develop this strategy into a standard tool for managing brain health in our older-age populations. The core strengths of INHANCE include offering an online brain training program that represents a low-risk, nonpharmaceutical approach that complements existing conventional methods for maintaining cognitive health in aging adults. In addition, it facilitates rapid, scalable global access for anyone with an internet-connected device.

Future research should evaluate the use of FEOBV-PET as a diagnostic tool for identifying individuals at risk for cognitive decline, its predictive value in clinical populations, and its relevance as an endpoint in interventional trials. In addition, it should establish the mechanistic foundation for pivotal trials that assess the role of brain training in preventing and treating mild cognitive impairment and dementia.

## Appendix

Multimedia Appendix 1 Peer-review report by the Center for Scientific Review Special Emphasis Panel - Center for Scientific Review - Small Business: Psycho/Neuropathology Lifespan Development, STEM Education (National Institutes of Health, USA).

## Abbreviations

ACTIVE: Advanced Cognitive Training for Independent and Vital Elderly
C-SSRS: Columbia Suicide Severity Rating Scale
DARTEL: Diffeomorphic Anatomical Registration Through Exponentiated Lie Algebra
FEOBV: 18F-fluoroethoxybenzovesamicol
GDS-SF: Geriatric Depression Scale – Short Form
INHANCE: Improving Neurological Health in Aging via Neuroplasticity-based Computerized Exercise
ITT: intent-to-treat
LORIS: Longitudinal Online Research and Imaging System
MoCA: Montreal cognitive assessment
MRI: magnetic resonance imaging
NIH EXAMINER: National Institutes of Health Executive Abilities: Measures and Instruments for Neurobehavioral Evaluation and Research
PET: positron emission tomography
ROI: regions of interest
V0: consent and screening visit
V1: baseline visit
V2: posttest visit
V3: follow-up visit (End of study)
WM: white matter

## Data Availability

The data sets generated and analyzed during this study are available in the LORIS repository 2 years after the completion of the trial [49].
