# Supplementary material for: Improving Neurological Health in Aging Via Neuroplasticity-Based Computerized Exercise: Protocol for a Randomized Controlled Trial
Source: JMIR Res Protoc. 2024 Aug 8;13:e59705. doi: 10.2196/59705 (PMC11342015; doi:10.2196/59705)
Supplement: Multimedia Appendix 1 [file resprot_v13i1e59705_app1.pdf]

SUMMARY STATEMENT

PROGRAM CONTACT: (Privileged Communication) *Release Date:* 07/12/2019

Revised Date:

[redacted]

Application Number: 2 R44 AG039965-05

Principal Investigators (Listed Alphabetically):

[redacted] (Contact)

Applicant Organization: POSIT SCIENCE CORPORATION

Review Group: ZRG1 RPHB-Y (12)
Center for Scientific Review Special Emphasis Panel
Small Business: Psycho/Neuropathology Lifespan Development, STEM Education

Meeting Date: 06/27/2019
Council: AUG 2019
Requested Start: 12/01/2019
RFA/PA: PA18-573
PCC: 3DCATMW

Project Title: Improving Neurological Health in Aging via Neuroplasticity-based Computerized Exercise (INHANCE)
SRG Action: Impact Score:42
Next Steps: Visit https://grants.nih.gov/grants/next_steps.htm
Human Subjects: 30-Human subjects involved - Certified, no SRG concerns
Animal Subjects: 10-No live vertebrate animals involved for competing appl.
Gender: 1A-Both genders, scientifically acceptable
Minority: 1A-Minorities and non-minorities, scientifically acceptable
Age: 7A-Only Adults, scientifically acceptable

Project Year	Direct Costs Requested	Estimated Total Cost
5	[redacted]	[redacted]
6		
TOTAL	[redacted]	[redacted]

ADMINISTRATIVE BUDGET NOTE: The budget shown is the requested budget and has not been adjusted to reflect any recommendations made by reviewers. If an award is planned, the costs will be calculated by Institute grants management staff based on the recommendations outlined below in the COMMITTEE BUDGET RECOMMENDATIONS section.

BUDGET MODIFICATIONS

2R44AG039965-05 [redacted]

COMMITTEE BUDGET RECOMMENDATIONS

RESUME AND SUMMARY OF DISCUSSION: This significant Phase IIB SBIR application proposes to conduct a clinical trial evaluation of INHANCE, a computerized cognitive training program based on neuroplasticity principles that is targeted to older adults in order to reduce long-term risk of dementia. Reviewers were moderately enthusiastic about the application, noting the significance and need for this type of study. The investigative team, led by multiple principal investigators, Thomas Van Fleet and Mouna Attarha, are exceptionally-qualified and highly experienced to conduct this Phase IIB clinical trial. The small business, Posit Science Corporation, provides a supportive environment to carry out the randomized clinical trial. Reviewers described the existing product as one of the leaders in the field, and that the plan to conduct a double-blind study is rigorous and well-justified. Along with these notable strengths, the review panel also identified some weaknesses that tempered their assessment of the application's potential impact. The findings from the prior research may be generalizable to a small segment of the older adult population without impairment, and although the proposed study will expand this, the sample size of 100 participants may not be large enough to detect clinical effectiveness. Moreover, whether the changes measured by the study will translate into real world cognitive changes and benefits is another concern. With regard to the study design, the control group will receive a Sudoku and Solitaire intervention that reviewers commented would not be comparable to the INHANCE treatment condition in terms of time and attention. As a Phase IIB, the application does not include letters of support from third parties who would show commercial potential and interest. Overall, the review panel reached consensus of a very solid application with a strong design and approach; however, the strengths only somewhat outweigh the weaknesses, leading to a predicted impact in the moderate range.

DESCRIPTION (provided by applicant): Advancing age is often accompanied by a decline of key neuromodulatory centers of the brain that support adaptive neuroplasticity. Deterioration of the underlying neurophysiology of the brain predisposes the individual to pathological and age-related cognitive decline. We propose to deploy a computerized cognitive training program (INHANCE) developed via two successful SBIR-funded phase II programs (R44AG039965; R44NS071780). The program will combine two previously validated, but historically independent, exercises that based on prior research may interact to reduce the long-term risk of dementia onset. We shall conduct a double-blind, parallel-arm, placebo-controlled, randomized clinical trial that will assess the short- and long-term efficacy of the program to improve neuromodulatory status (via positron-emission tomography) and neuropsychological outcomes (via standard assessment measures). The broader objectives of the project are two-fold. One is to address the rapid rise in neurodegenerative disease and the other is to shed light on the underlying neuromodulatory mechanism(s) through which certain forms of cognitive training achieve neuropsychological improvement. An effective, low-cost, and highly-scalable program available on the most popular internet-connected devices offers global reach and the possibility of extending the cognitive health of older adults. With effective computerized tools in hand we can enrich the lives of patients and their families, alleviate the enormous financial cost associated with minor and major forms of impairment, and progress swiftly toward the goal of creating a healthier society and a more efficient healthcare system.

PUBLIC HEALTH RELEVANCE: The goal of the current Phase IIB proposal is to evaluate the efficacy of a neuroplasticity-based, computerized cognitive training program—INHANCE (Improving Neurological Health in Aging via Neuroplasticity-based Computerized Exercise)—that trains speed of processing and attentional control in healthy older adults. This web-delivered program aims to improve cognition, as evidenced by standard neuropsychological measures, and plasticity-based cholinergic function as measured through positron-emission tomography. These outcomes are widely applicable to

the cognitive health and life- quality of elderly Americans, and have further potential to delay degenerative processes observed in normal aging.

CRITIQUE 1

Significance: 4
Investigator(s): 1
Innovation: 3
Approach: 4
Environment: 2

Overall Impact: In this Phase IIB Application, the investigators propose to carry out a RTC test of the ability of their Brain Training Program (INHANCE) to improve cognitive performance in "normal" aging individuals (>65). Specifically, they propose to test changes in ACH in the forebrain that are coincident with the cognitive changes that they predict will follow the training. Clearly this is an important area, and one that has been confusing in the current literature. While many products are available that claim to improve cognition through gaming type activities, none of the current data on their benefits is totally convincing, and much of the available data supporting the claims of companies has been very weak, mostly carried out via in-house "experiments." The applicant organization is clearly separating itself from these other companies. It has a very strong neuroscience support with Dr. Merzenich and the strongest current study of cognitive training programs was done on its product ACTIVE. The authors clearly lay out weaknesses in the past studies of their own and others, and lay out a reasonable RTC approach. Much of the proposed study is strong, including the Stats, leadership and DSMB. However, there are some weaknesses in the proposal that lessen enthusiasm. First, the study is directed almost entirely by the company leaders. This has been a major problem in the past, with company-led research often giving an appearance of non-objectivity. Clearly, an outside evaluation of the product would be more expensive, but also more convincing. Second, the controls are somewhat defined, but defined but may be somewhat weak in that solitaire and Sudoku are relatively common games and may be less than optimal, and it is not entirely clear that the product will effect a gain in cognitive performance, i.e., does it provide gains that are generalizable, especially in the target "normal" individuals. Overall the rational is strong and the product to be tested is very deserving, but weaknesses exist, and those weaknesses somewhat balance the strengths.

1. Significance:

Strengths

- The world needs critical tests of these types of products, so that ones that are truly generalizable in their abilities can begin to improve cognitive health is an ageing society.
- The team at the company is strong.
- The product to be tested has the strongest test results of all similar products.

Weaknesses

- Having the company totally direct research on its own products will leave many concerned about bias.
- While the ACTIVE study was positive and stronger than other programs that were tested critically, it showed relatively weak cognitive effects.
- Moving to testing neuromodulator pathways, before there is clear evidence that the product will improve generalized cognitive function may be problematic. Increased forebrain ACH does not necessarily denote that cognition will be improved.

- The dose-dependency and the benefits of six hours of treatment is interesting but not yet very complete. But the real goal at this point appears more important to demonstrate that INHANCE can blunt the progression of memory decline, requiring strong effectiveness research.

2. Investigator(s):

Strengths

- Dr. Merzenich is an outstanding neuroscientist.
- The other company leaders are very committed to developing an affective product that will improve cognitive abilities.

Weaknesses

- None noted.

3. Innovation:

Strengths

- This is a strong RCT, something that has not been carried out by other companies with similar products**.**

Weaknesses

- None noted.

4. Approach:

Strengths

- The RCT is well design in most of its aspects.
- The mechanistic studies are excellent in their scale and approach. We will learn more about ACH from these studies.
- The studies developed out of very successful phase I STTR research studies.
- Even if the games do not show a generalized benefit to cognition, they may be very effective in other areas, e.g., improved focus.

Weaknesses

- The applicants posit that four 30m sessions per week for 10 weeks will be sufficient to improve cognition, this is not well defended.
- Nearly all of the positive reports on which the study is based are authored by company personnel.
- It is unclear how strong the active control will be and whether a passive control is also needed.
- African-Americans appear to be underrepresented in the study, albeit, it is in Canada.

5. Environment:

Strengths

- The environment is excellent.

Weaknesses

- None noted.

Study Timeline:

Strengths

- The timeline is very reasonable.

Weaknesses

- None noted.

Phase II (Type 2 R42 and Type 2 R44 applications):

Not Applicable

Direct Phase II (Type 1 R44 applications See Face Page):

Acceptable

- This is a reasonable follow up on a success pair of Phase I studies.

Protections for Human Subjects:

Acceptable Risks and Adequate Protections

- This appears to be very good, albeit there is a relative lack of African-Americans.

Data and Safety Monitoring Plan (Applicable for Clinical Trials Only):

Not Applicable (No Clinical Trials)

Inclusion Plans:

- Sex/Gender: Distribution justified scientifically
- Race/Ethnicity: Distribution justified scientifically
- For NIH-Defined Phase III trials, Plans for valid design and analysis: Not applicable
- Inclusion/Exclusion Based on Age: Distribution justified scientifically
- This is all well designed.

Vertebrate Animals:

Not Applicable (No Vertebrate Animals)

Biohazards:

Not Applicable (No Biohazards)

Budget and Period of Support:

Recommend as Requested

CRITIQUE 2

Significance: 2
Investigator(s): 2
Innovation: 4
Approach: 4
Environment: 1

Overall Impact: This is an application for a Phase IIB Continuing Renewal to evaluate the efficacy and neurobiological mechanisms of a computerized cognitive training program for older adults. The training is a combination of two previously tested but separate programs, one of which was developed via two SBIR Phase II projects. Progress and published research from this team toward meeting Phase IIB objectives from these prior Phase II studies provides a solid foundation for this renewal application. Merging two possibly synergistic cognitive training programs is an incremental improvement to existing training products on the market for age-related cognitive decline. The results might offer new insights into neural mechanisms of change or neuroprotection against dementia. This strong and experienced team proposes a high-powered RCT that will address many of the weaknesses in the rigor of prior research in this area. The commercial plan also provides robust evidence of the training program's potential value to the scientific community and benefit to end-user consumers, as well as steps to market and plug into healthcare systems for older adults. However, the commercial finance plan does not include considerable partners interested in the venture. This is a glaring weakness in terms of what is expected in a Phase IIB commercialization plan. It is also not clear how the benchmark to determine adequate dosage for full therapeutic benefit (Aim 2) will be calculated or fleshed out. Nevertheless, this a strong and well-done application where the strengths outweigh the weaknesses, making for an impact in the high range. Human subjects protection, gender considerations, ethnicity distributions, and age inclusion are all justified and appropriate.

1. Significance:

Strengths

- Non-pharmacological interventions to mitigate dementia risk is an area of high priority for NIH and many health agencies all over the world. The application addresses a very important problem that is being studied at every level of science, from basic to community treatment to national policies.
- Evidence from prior published research on UFOVt from this team and others in the field support the goals of this Continuing Renewal.
- Prior Phase II aims and results, quantitative milestones on efficacy, and achieved targeted planned enrollment from the two SBIR Phase II studies are clearly outlined. The summaries highlight the overall progress of their intervention for age-related cognitive decline and the importance of their findings in advancing scientific knowledge of preventive paradigms.
- The commercial plan provides robust evidence of the training program's potential value to the scientific community and benefit to end-user consumers. The clear and well-reasoned explanations of the competitive market and steps for FDA regulatory clearance all contribute to the likelihood of commercial success.

Weaknesses

- The findings on dose dependency of UFOVt (each hour producing 10% lower hazard for dementia) and the complementary benefits of six hours of TAPAT+UFOVt--while promising--should be extrapolated with caution. While INHANCE may end up being a scalable and possibly efficacious cognitive training program, the goal to market (and seek FDA approval) this product as a "preventative" treatment is beyond the scope of IIB efficacy and neurobiological testing. Effectiveness trials are needed to make such conclusions.

2. Investigator(s):

Strengths

- PI Van Vleet brings a wealth of experience in SBIR projects in similar areas, with the proven ability to organize and manage all aspects of a Phase IIB project.
- His team has the appropriate experience in clinical trial coordination and management, with appropriate expertise in PET ligands and neuroimaging analyses.
- The team continues to demonstrate a high record of accomplishments on this topic. Co-I Merzenich is a preeminent scientist and pioneer in this area.

Weaknesses

- [redacted]

3. Innovation:

Strengths

- Merging two possibly synergistic or additive cognitive training programs is an incremental change to existing training products on the market for age-related cognitive decline.
- The results might offer new insights into neural mechanisms of change or neuroprotection against dementia.
- Possible new capabilities from this merger will refine the understanding of core executive control functions and processing speed as valid targets.

Weaknesses

- The project does not necessarily employ novel theoretical concepts that will shift clinical practice.
- While this proposal (and other studies in aging from the team) are of high quality, the proposed "completely new directions for research" is a bit overstated. This line of inquiry in biological brain health is not new and other commercially available brain-training programs offer similar training paradigms.

4. Approach:

Strengths

- The overall approach and methodology are sound and offer a rigorous strategy to test and establish the product's efficacy.
- The adequately powered RCT that includes frequent interval benchmarks, proxy and distal outcome, dose response, and effect duration will address many of the weaknesses in the rigor of prior research in this area.
- Milestones achieved in the past Phase II projects, alternative strategies in case of potential problems in attrition, technical requirements, adherence, and result interpretation are all clearly outlined.

- The commercialization plan provides a thorough description of Phase III commercialization efforts including how the product can be plugged into healthcare systems for older adults and steps to seek FDA approval for prescription control. These two matters will significantly impact commercialization.

Weaknesses

- In terms of what is expected in a Phase II commercialization plan, letters of support to back the finance plan are necessary. There is only one letter for this project and the stated support is nominal. At this late stage of product development, the commercial finance plan should include considerable partners interested in the venture.
- It is not clear how the benchmark to determine adequate dosage for full therapeutic benefit (Aim 2) will be calculated or fleshed out.

5. Environment:

Strengths

- MNI and PSC together have the capabilities and resources to carry out the project. They have a proven record of conducting similar efficacy trials in older adults.

Weaknesses

- None noted.

Study Timeline:

Strengths

- Start-up will be minimal (if at all) since all personnel, equipment, and the enrollment infrastructure are already in place from prior Phase II projects.
- The projected timeline is feasible and justified and based on successful Phase II timelines.
- Potential challenges to attrition and adherence are addressed.

Weaknesses

- None noted.

Phase II (Type 2 R42 and Type 2 R44 applications):

Acceptable

- Progress toward meeting Phase IIB objectives from prior Phase II studies provides a solid foundation for this renewal application.

Protections for Human Subjects:

Acceptable Risks and Adequate Protections

- Protection of older adults is adequate and justified

Data and Safety Monitoring Plan (Applicable for Clinical Trials Only):

Acceptable

- DSMP is adequate.

Inclusion Plans:

- Sex/Gender: Distribution justified scientifically
- Race/Ethnicity: Distribution justified scientifically
- For NIH-Defined Phase III trials, Plans for valid design and analysis: Not applicable
- Inclusion/Exclusion Based on Age: Distribution justified scientifically
- Gender, ethnicity distributions, and age inclusion are all justified and appropriate.

Vertebrate Animals:
Not Applicable (No Vertebrate Animals)

Biohazards:
Not Applicable (No Biohazards)

Phase II B Competing Renewals:

- This project continues Phase II product development by refining the intervention in preparation for commercialization. If the aims are achieved, there is a high probability of commercial success and rapid market share penetration. While the importance of achieved milestones through prior SBIR studies are included, investment by 3rd parties is noticeably lacking for a Phase IIB application.

Resource Sharing Plans:
Acceptable

Authentication of Key Biological and/or Chemical Resources:
Acceptable

Budget and Period of Support:
Budget Modifications Recommended (in amount/time)
Recommended budget modifications or possible overlap identified:

- [redacted]

CRITIQUE 3

Significance: 4
Investigator(s): 1
Innovation: 4
Approach: 1
Environment: 1

Overall Impact: This R44 Phase IIB aims to deploy a computerized cognitive training program (INHANCE) developed via two previously successful SBIR-funded phase II programs (R44AG039965;

R44NS071780). The project proposes to combine these historically independent products and conduct a double-blind, parallel-arm, placebo-controlled, randomized clinical trial to assess the efficacy in neuromodulatory (PET) and neuropsychological outcomes. This project is supported by a strong existing scientific foundation and is being undertaken by a research team that has collaborated effectively together multiple times in the past. The innovative nature of this project includes the potential for demonstrating underlying neurobiological changes associated with a targeted regimen of cognitive training. The enthusiasm is dampened by the concern that demonstration of neurobiological changes in the absence of generalizable real-world outcomes does not represent a strong innovation in this area. Further, the use of two tasks is a challenge to the significance of this project given the concern for real-world transfer. The approach to this pivotal RCT is strong and the potential for impact is moderate.

1. Significance:

Strengths

- Pathological cognitive decline is a growing global problem for which there are no disease modifying agents.
- The underlying scientific rationale for the proposed work is strong and based on empirical neurobiological study.

Weaknesses

- While cognitive training has a body of evidence supporting its role as a single-domain intervention to improve cognitive health, a multi-domain approach to non-pharmacological interventions, including cognitive training, are increasingly recognized.
- The potential for showing increased acetylcholine associated with cognitive training does not replace the need to show generalizable cognitive gains associated with cognitive training.
- Utilizing a subset of tasks (TAPAT and Double Decision) is a further challenge for demonstrating generalizability of cognitive training to real world performance.

2. Investigator(s):

Strengths

- The Co-PIs have significant research and clinical experience germane to the proposed work.
- Site PI has the clinical expertise and experience to support this work.

Weaknesses

- No major weaknesses noted.

3. Innovation:

Strengths

- An effective, low-cost, and scalable product that is clinically validated to improve cognitive health in older adults is innovative.

Weaknesses

- Further evidence is needed in demonstrating computerized cognitive training as a single-domain intervention that is effective for generalizable cognitive gains and risk reduction for pathological cognitive decline.

- While a demonstration of neuromodulatory mechanisms of action associated with cognitive training would provide marketing strength for commercialization, it does not represent innovation with respect to computerized cognitive training.
- The INHANCE method would provide a potential dose-response regimen for the population of interest, but there is concern that demonstration of changes in acetylcholine associated (or not) with changed in cognitive task performance without generalizable real world task performance is redundant with existing bodies of evidence.

4. Approach:

Strengths

- A strong approach is provided for the extension of previous Phase II work in a rigorously designed pivotal RCT.
- A strong commercialization plan is presented, addressing contingencies and forward thinking with respect to FDA clearance.

Weaknesses

- No major weaknesses noted.

5. Environment:

Strengths

- Adequate justification for involvement of project activities outside of the US (McGill university) is provided.
- Previous collaborations strengthen the current proposal.
- The Company facilities, equipment and environment are appropriate for the proposed projects.
- Trial site has access to the required subjects

Weaknesses

- No major weaknesses noted.

Study Timeline:

Strengths

- Study timeline provides adequate time for participant recruitment, enrollment, and data collection.

Weaknesses

- None noted.

Phase II (Type 2 R42 and Type 2 R44 applications):

Acceptable

- This is an appropriate extension of already completed Phase II work with high potential for commercializability. Existing results and progress from completed Phase II work has been demonstrated.

Protections for Human Subjects:

Acceptable Risks and Adequate Protections

- Appropriate and thorough

Data and Safety Monitoring Plan (Applicable for Clinical Trials Only):
Acceptable

- Very thorough.

Inclusion Plans:

- Sex/Gender: Distribution justified scientifically
- Race/Ethnicity: Distribution justified scientifically
- For NIH-Defined Phase III trials, Plans for valid design and analysis: Not applicable
- Inclusion/Exclusion Based on Age: Distribution justified scientifically
- Healthy older adults between 65-85 years old given the population of interest.

Vertebrate Animals:
Not Applicable (No Vertebrate Animals)

Biohazards:
Not Applicable (No Biohazards)

Phase II B Competing Renewals:

- This is an appropriate extension of already completed Phase II work with high potential for commercializability. Existing results and progress from completed Phase II work has been demonstrated.

Select Agents:
Not Applicable (No Select Agents)

- The PET ligand used in this trial, FEOBV, is not classified as a select agent or toxin by the HHS or USDA (7CFR Part 331, 9 CFR Part 121, and 42 CFR Part 73).

Budget and Period of Support:
Recommend as Requested

THE FOLLOWING SECTIONS WERE PREPARED BY THE SCIENTIFIC REVIEW OFFICER TO SUMMARIZE THE OUTCOME OF DISCUSSIONS OF THE REVIEW COMMITTEE, OR REVIEWERS' WRITTEN CRITIQUES, ON THE FOLLOWING ISSUES:

PROTECTION OF HUMAN SUBJECTS: ACCEPTABLE

INCLUSION OF WOMEN PLAN: ACCEPTABLE

INCLUSION OF MINORITIES PLAN: ACCEPTABLE

INCLUSION ACROSS THE LIFESPAN PLAN: ACCEPTABLE

COMMITTEE BUDGET RECOMMENDATIONS:
[redacted]

Footnotes for 2 R44 AG039965-05; PI Name: [redacted]

NIH has modified its policy regarding the receipt of resubmissions (amended applications). See Guide Notice NOT-OD-14-074 at http://grants.nih.gov/grants/guide/notice-files/NOT-OD-14-074.html. The impact/priority score is calculated after discussion of an application by averaging the overall scores (1-9) given by all voting reviewers on the committee and multiplying by 10. The criterion scores are submitted prior to the meeting by the individual reviewers assigned to an application, and are not discussed specifically at the review meeting or calculated into the overall impact score. Some applications also receive a percentile ranking. For details on the review process, see http://grants.nih.gov/grants/peer_review_process.htm#scoring.

MEETING ROSTER

Center for Scientific Review Special Emphasis Panel
CENTER FOR SCIENTIFIC REVIEW
Small Business: Psycho/Neuropathology Lifespan Development, STEM Education

ZRG1 RPHB-Y (12)
06/27/2019 - 06/28/2019

Notice of NIH Policy to All Applicants: Meeting rosters are provided for information purposes only. Applicant investigators and institutional officials must not communicate directly with study section members about an application before or after the review. Failure to observe this policy will create a serious breach of integrity in the peer review process, and may lead to actions outlined in NOT-OD-14-073 at https://grants.nih.gov/grants/guide/notice-files/NOT-OD-14-073.html and NOT-OD-15-106 at https://grants.nih.gov/grants/guide/notice-files/NOT-OD-15-106.html, including removal of the application from immediate review.

CHAIRPERSON(S)

[redacted]

MEMBERS

[redacted]

[redacted]

[redacted]

[redacted]

[redacted]

[redacted]

[redacted]

[redacted]

[redacted]

[redacted]

[redacted]

[redacted]

[redacted]

[redacted]

[redacted]

[redacted]

[redacted]

[redacted]

[redacted]

[redacted]

[redacted]

[redacted]

[redacted]

[redacted]

[redacted]

[redacted]

[redacted]

[redacted]

[redacted]

[redacted]

[redacted]

[redacted]

[redacted]

[redacted]

[redacted]

[redacted]

[redacted]

[redacted]

SCIENTIFIC REVIEW OFFICER

[redacted]

EXTRAMURAL SUPPORT ASSISTANT

[redacted]

Consultants are required to absent themselves from the room during the review of any application if their presence would constitute or appear to constitute a conflict of interest.
